# Nonadhesive Culture System as a Model of Rapid Sphere Formation with Cancer Stem Cell Properties

**DOI:** 10.1371/journal.pone.0031864

**Published:** 2012-02-16

**Authors:** Su-Feng Chen, Yun-Ching Chang, Shin Nieh, Chia-Lin Liu, Chin-Yuh Yang, Yaoh-Shiang Lin

**Affiliations:** 1 Department of Dental Hygiene, China Medical University, Taichung, Taiwan; 2 Graduate Institute of Life Sciences, National Defense Medical Centre, Taipei, Taiwan; 3 Department of Pathology, National Defense Medical Centre and Tri-Service General Hospital, Taipei, Taiwan; 4 Department of Dentistry, National Defense Medical Center and Tri-Service General Hospital, Taipei, Taiwan; 5 Department of Otolaryngology-Head and Neck Surgery, National Defense Medical Centre and Tri-Service General Hospital, Taipei, Taiwan; National Cancer Institute, United States of America

## Abstract

**Background:**

Cancer stem cells (CSCs) play an important role in tumor initiation, progression, and metastasis and are responsible for high therapeutic failure rates. Identification and characterization of CSC are crucial for facilitating the monitoring, therapy, or prevention of cancer. Great efforts have been paid to develop a more effective methodology. Nevertheless, the ideal model for CSC research is still evolving. In this study, we created a nonadhesive culture system to enrich CSCs from human oral squamous cell carcinoma cell lines with sphere formation and to characterize their CSC properties further.

**Methods:**

A nonadhesive culture system was designed to generate spheres from the SAS and OECM-1 cell lines. A subsequent investigation of their CSC properties, including stemness, self-renewal, and chemo- and radioresistance *in vitro*, as well as tumor initiation capacity *in vivo*, was also performed.

**Results:**

Spheres were formed cost-effectively and time-efficiently within 5 to 7 days. Moreover, we proved that these spheres expressed putative stem cell markers and exhibited chemoradiotherapeutic resistance, in addition to tumor-initiating and self-renewal capabilities.

**Conclusions:**

Using this nonadhesive culture system, we successfully established a rapid and cost-effective model that exhibits the characteristics of CSCs and can be used in cancer research.

## Introduction

Oral squamous cell carcinoma (OSCC) is one of the most common and lethal head and neck malignancies in Taiwan and worldwide [1], [2]. OSCC is a disease that is difficult to treat because of the diverse treatment strategies available and the variable natural behavior of the cancer. Local invasion and frequent regional lymph node metastases, together with relative resistance to chemotherapeutic drugs, lead to an unpredictable outcome [2]–[4]. Despite increased experience in surgical technology and adjuvant treatments, the overall prognoses of OSCC remain unimproved, resulting in the urgent need of a novel strategy for OSCC treatment [3], [5].

Substantial evidences from recent studies show that solid tumors contain a subpopulation of cancer stem cells (CSCs) [6]–[8]. It is well known that CSCs play an important role in tumor initiation, progression, metastasis, and therapeutic resistance [9]–[11]. However, the putative CSCs from OSCC have not been well characterized. It is hypothesized that CSCs possess several characteristics that make them resistant to conventional chemo- and radiotherapy, including high expression of drug transporters, relative cell-cycle quiescence, high function of DNA repair machinery, and resistance to apoptosis [12], [13]. The identification and characterization of CSCs from OSCC are crucial for facilitating the monitoring, treatment, and prevention of the disease.

The isolation of CSCs from cancer cells has been achieved successfully via the use of different techniques. The isolation of CSCs is performed using flow cytometry based on the expression of specific cell surface markers, such as CD133, CD44 and ALDH1, by CSCs [14]–[20]. Because of the therapeutic resistance of CSCs, sorting the side populations of cancer cells via intracellular Hoechst 33342 exclusion or selecting chemotherapeutic-drug-resistant cells has also been used for the identification and characterization of CSCs [21]–[23]. Concurrent studies confirmed that the sphere culture system is as efficient in separating CSCs from many solid tumors or cancer cells lines. These studies have suggested that CSCs can be enriched in spheres when these are cultured in serum-free medium supplemented with adequate mitogens, such as the basic fibroblast growth factor (bFGF) and epidermal growth factor (EGF) [11], [24]–[26]. However, the derivation of CSCs from solid tumors and cancer cell lines cultured in serum-free medium supplemented with bFGF and EGF is a time-consuming process and 2–6 weeks are needed for sphere formation [11], [24]–[26]. Furthermore, the selected growth factors, such as the platelet-derived growth factor, bFGF, and EGF, are costly and ineffective. To overcome these drawbacks and limitations, we used a purpose-designed nonadhesive sphere culture system to identify and enrich CSCs from established human OSCC cell lines, and to characterize their CSC properties further using phenotypic/genotypic characterization.

## Results

### The sphere formation from human OSCC cell lines

**Figure 1:**
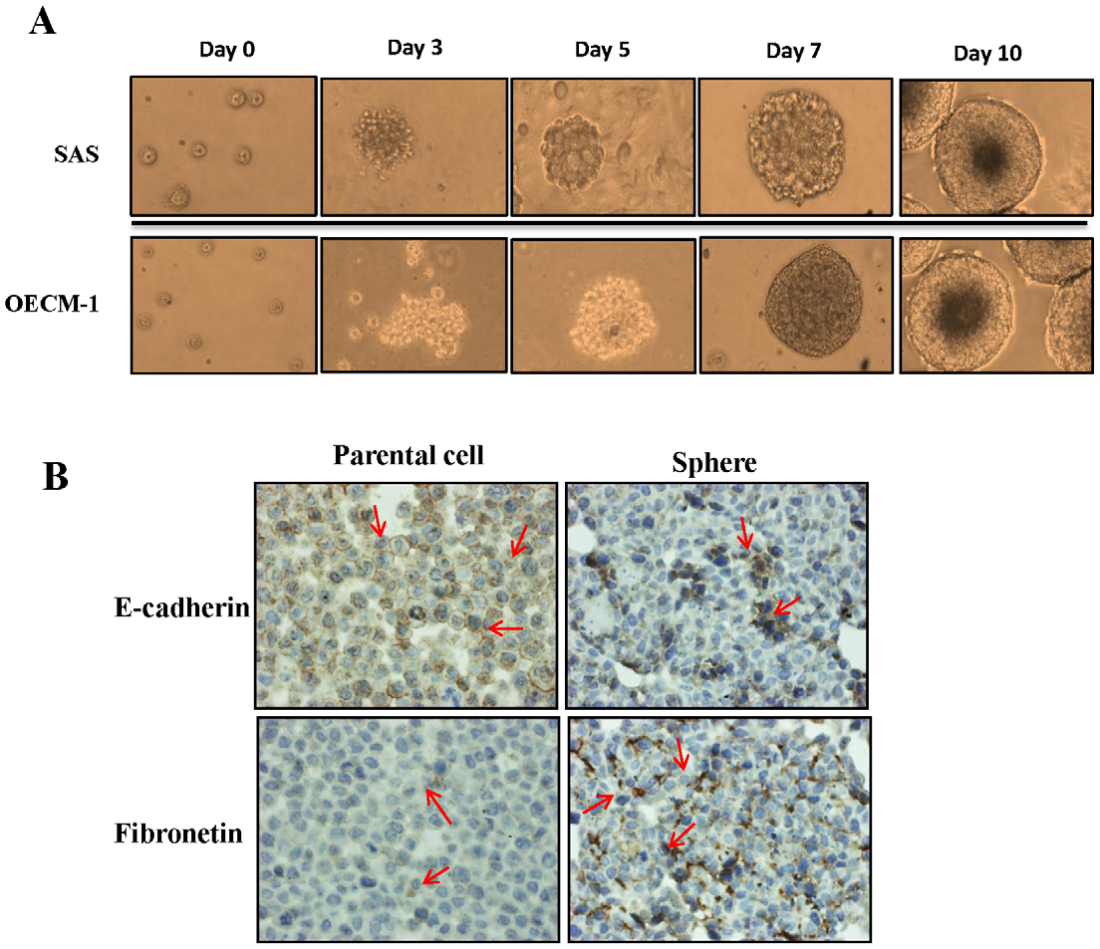
The sphere formation from human OSCC cell lines. (A) Phase-contrast photomicrographs of the spheres cultured from SAS (top) and OECM-1 (bottom) cell lines using a nonadhesive design (four leftmost upper and lower panels: from day 0 to day 7, magnification, 200×; and rightmost upper lower panels: day 10, magnification, 100×). (B) Immunohistochemistry results showing diverse expression patterns of representative epithelial–mesenchymal transition (EMT) markers in OECM1 parental cells and spheres (magnification, 200×).

OSCC cell lines (SAS and OECM-1) were gently dissociated into single cells and seeded into culture plastic wares with a nonadhesive coating, as shown in Figure 1. Part of the suspension of cells may undergo apoptosis during the first 2 days when cultured in a nonadhesive, suspended environment. Some of the suspended cells aggregated and then merged and differentiated into three-dimensional (3D) balls with a spheroid configuration. The subsequent morphological alteration (∼3–5 days) consisted of floating spheres. After 5–7 days of culture, spheres with a round and smooth contour were observed. These spheres grew gradually over time (Figure 1A). Morphologically, the spheres appeared more tightly attached, clustering or overlapping in a 3D configuration, compared with those observed in the parental cells. One previous study suggested that the derivation of spheres from cancer cell lines or primary culture cells may accompany the alteration of phenotypic/genotypic characteristics, such as the epithelial–mesenchymal transition (EMT) [27]. The representative EMT markers E-cadherin and fibronectin were chosen to identify, and compare the differences between, the parental cells and spheres in OECM-1 and SAS cells. Microscopic examination of immunohistochemically stained parental cells and spheres showed the presence of generalized and diffuse expression of E-cadherin and sparse expression of fibronectin in parental cells, whereas spheres exhibited loss of expression of E-cadherin and overexpression of fibronectin (Figure 1B).

### Expression of putative stem cell surface markers

**Figure 2:**
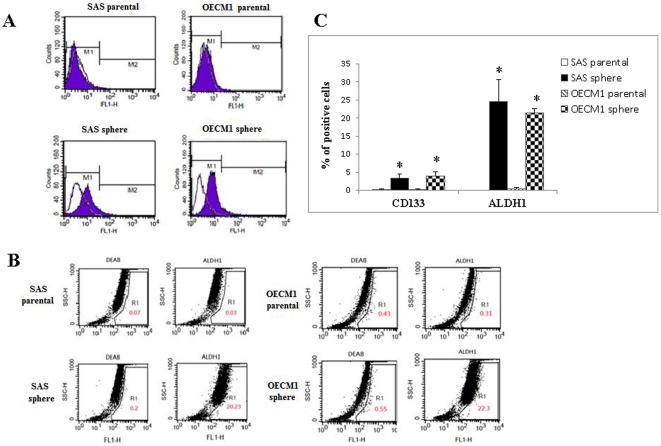
Comparison of the expression of specific surface markers of CSCs between parental cells and spheres. (A) The parental cells and spheres were either stained with a negative-control IgG antibody (open space) or anti-CD133 experimental antibodies (solid space). (B) Comparison of the expression of ALDH1 between parental cells and spheres; DEAB, an inhibitor of ALDH1, was used as a negative control. (C) Quantitative and statistical comparisons of the percentage of positive signals for CD133 and ALDH1 between parental cells and spheres (*P<0.05).

To elucidate whether spheres could enrich cells expressing putative cancer stem cell markers, we chose to analyze the expression profile of two representative stem cell surface markers of OSCC, CD133 and ALDH1 [11], [14]–[18]. As shown in Figure 2A and B, the parental cells and spheres (after 7 days of nonadhesive culture) were positively stained for CD133 and ALDH1. Expression of CD133 and ALDH1 was usually absent or very low in parental cells. We detected a 3–4% increase in CD133 expression and a 20–30% increase in ALDH1 expression in spheres compared with parental cells. The levels of expression of CD133 and ALDH1 were significantly higher in spheres than they were in parental cells (Figure 2C).

### Expression of cancer stem cell genes and related proteins

**Figure 3:**
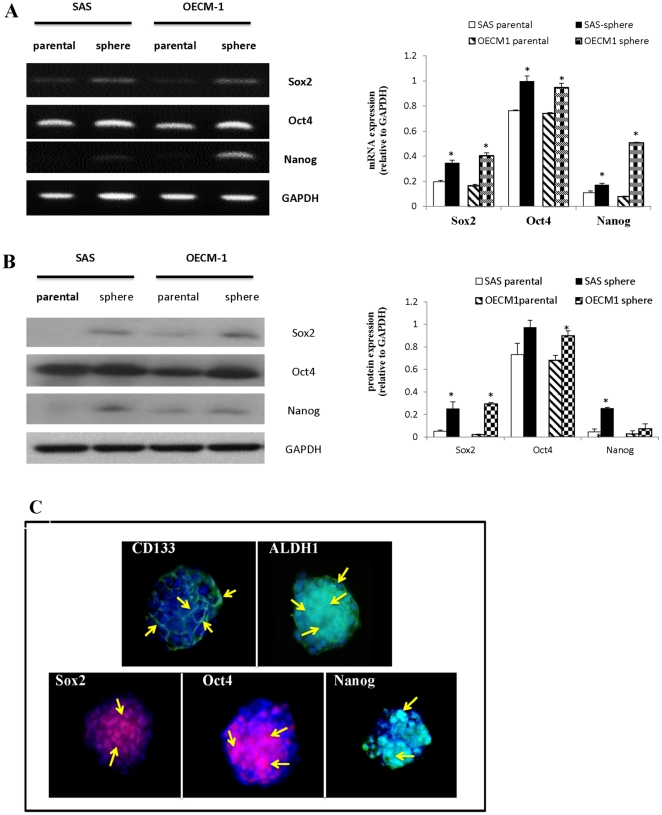
Comparison of the expression of CSC markers between parental cells and spheres. (A) A RT-PCR analysis showed that the expression of the *SOX2, Oct4, and NANOG* genes was upregulated in spheres compared with parental cells. (B) Western blotting analysis showed that the expression of SOX2, Oct4, and NANOG was upregulated in spheres compared with parental cells. (C) Immunofluorescence analysis of CSC markers in spheres demonstrated the presence of CSCs with variable levels of expression of CD133, ALDH1, SOX2, Oct4, and NANOG, as indicated by the arrows (magnification, 200×).

The expression of stem cell genes and related proteins, including *SOX2*, *Oct4*, and *NANOG*, was examined at the transcriptional and translational levels. Total RNA was purified from parental cells and spheres after 7 days of nonadhesive culture. The levels of SOX2, Oct4, and NANOG transcripts were significantly increased in spheres compared with parental cells, as assessed using reverse transcription PCR analysis (Figure 3A). Western blotting data showed that the expression of the SOX2, Oct4, and NANOG proteins was also upregulated in spheres compared with parental cells (Figure 3B). Furthermore, we used immunofluorescence staining to assess the cellular levels of CD133, ALDH1, SOX2, Oct4, and NANOG in spheres. We observed diverse expression patterns for these proteins, as indicated in Figure 3C, which suggests the heterogeneity of OSCC. CD133 was expressed in the cell membrane and ALDH1 was expressed in the cell membrane and cytoplasm, whereas SOX2, Oct4, and NANOG were expressed in the nucleus.

### Radio- and chemosensitivity

**Figure 4:**
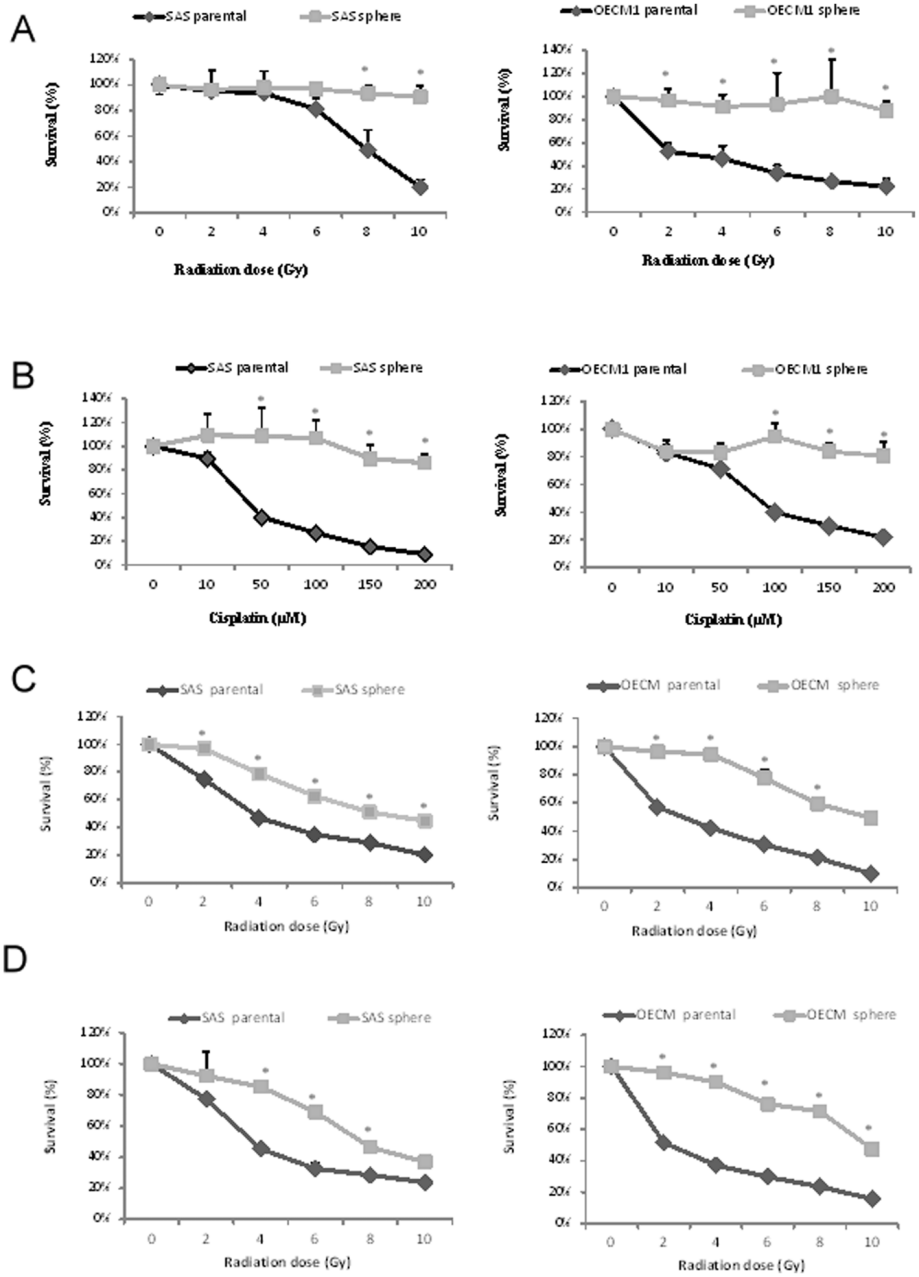
Comparison of radio- and chemosensitivity between parental cells and spheres of the two cell lines. Significant differences in (A) radiosensitivity and (B) chemosensitivity were observed between parental cells and spheres. (C) Combined chemo- and radiotherapy (CCRT) with initial chemotherapy for 24 h followed by radiation. (D) CCRT with initial radiation followed by chemotherapy for 24 h. The two CCRT regimens were more effective in reducing the survival rate of parental cells and spheres compared with single treatment using either radiation or chemotherapy (*P<0.05).

To assess the radiosensitivity of the parental cells and spheres, we treated these cells and spheres with radiation doses up to 10 Gy to evaluate cell viability, which was measured using an MTS assay after 36 h of radiation treatment. Spheres were more radioresistant than parental cells (Figure 4A). We also examined the chemosensitivity of the parental cells and spheres using cisplatin. Parental cells and spheres were treated with cisplatin for 48 h and cell viability was measured subsequently using an MTS assay (Figure 4B). Spheres were more resistant to cisplatin than parental cells. To imitate the clinical condition, we administered a combined chemo- and radiotherapy (CCRT) treatment, with (1) initial chemotherapy consisting of 20 µM cisplatin for 24 h followed by radiation (Figure 4C) or (2) initial radiation followed by chemotherapy using 20 µM cisplatin for 24 h (Figure 4D). The results of treatment using these two CCRT regimens revealed that the combinations were more effective in reducing the survival rate of the parental cells and spheres compared with single treatment of either radiation or chemotherapy. In addition, spheres were more resistant than the parental cells (with variable significance levels) when using the combined treatment.

### *In vivo* tumorigenicity

**Figure 5:**
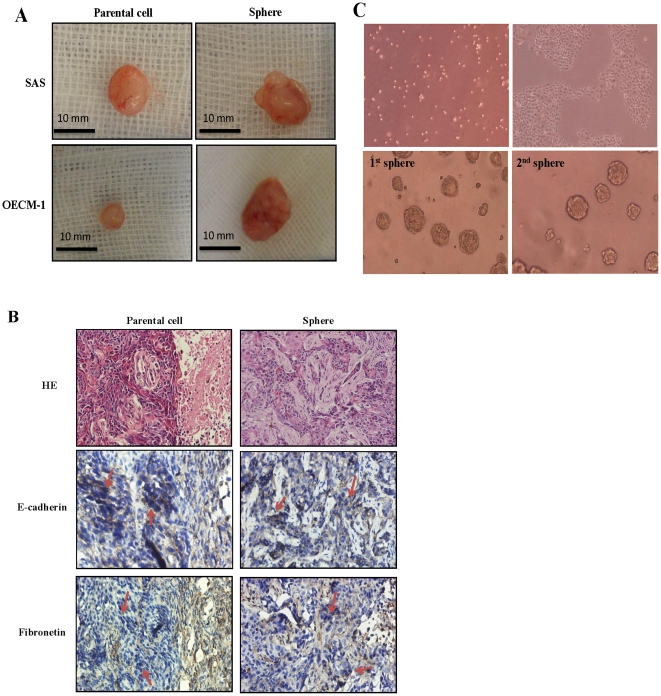
Comparison of newly generated tumors between OECM-1-derived parental cells and spheres in NOD/SCID mice. (A) Gross appearance of a representative tumor formed by inoculation of parental cells and dissociated spheres into NOD/SCID mice (n = 3 in each group). (B) Corresponding histological findings and immunohistochemical results for representative EMT markers in NOD/SCID mice (magnification, 100×). (C) Primary culture of dissociated cells from OECM-1-induced spheres originally isolated from NOD/SCID mice demonstrated a gradual transformation of primary (1^st^) and secondary (2^nd^) spheres (magnification, 100×).

To confirm the enriched tumor-initiating capabilities of spheres *in vivo*, both parental cells and spheres were injected into nude mice, for analysis of transplanted tumorigenicity. Spheres derived from SAS cells gave rise to tumors when 1×10^5^ cells were injected into mice (two out of three mice), and spheres derived from OECM-11 cells generated tumors when only 1×10^4^ cells were injected into mice (one out of three mice). In contrast, 1×10^6^ parental cells were needed to generate tumors, suggesting that spheres were enriched for tumor-initiating cells by at least 10- to 100-fold compared with parental cells (Table 1). A comparative analysis of gross appearance between the tumors newly generated from parental cells and spheres revealed the presence of significant differences regarding size and contour. Spheres yielded tumors of a much larger size with an irregular, expansible contour compared with the tumors generated by parental cells (Figure 5A). A comparative analysis of the corresponding histological and immunohistochemical results for representative EMT markers showed that tumors derived from spheres appeared to be more aggressive and have a mesenchymal-like appearance and prominent stromal invasion compared with the tumors derived from parental cells. We observed uneven expression of E-cadherin in tumors derived from parental cells and a loss of E-cadherin expression in tumors derived from spheres. There was an obvious overexpression of fibronectin in tumors derived from spheres compared with tumors derived from parental cells (Figure 5B). Primary cultures prepared from the resection of tumors induced by spheres in NOD/SCID mice demonstrated a gradual transformation of primary and secondary sphere formation, suggesting that spheres have a powerful capacity for self-renewal (Figure 5C).

**Table 1:** Tumorgenicity of the parental cells and spheres.

Cell line	SAS		OECM1	
Cell number for injection	parental cell	sphere	parental cell	sphere
10^4^	0/3	0/3	0/3	**1/3**
10^5^	0/3	**2/3**	0/3	**2/3**
10^6^	**2/3**	**3/3**	**3/3**	**3/3**

## Discussion

The concept of CSCs and their applications have been reported in recent decades. The term “cancer stem cell” was defined in 2006 by the American Association for Cancer Research Workshop on Cancer Stem Cells as a cell within a tumor that possesses the capacity to self-renew and to generate the heterogeneous lineages of cancer cells that comprise the tumor [6]. A review of the literature demonstrated that CSCs were first isolated by Bonnet and Dijk in acute myeloid leukemia, and Al Hajj was the first to identify them in solid tumors [28], [29]. To date, CSCs have been identified in many solid tumors, including brain, breast, lung, prostate, and colon cancers [24], [25], [30]–[33].The CSC theory clarifies not only the issue of tumor initiation, development, metastasis, and relapse, but also the ineffectiveness of conventional cancer therapies. According to current knowledge, the initiation, recurrence, and metastasis of cancers may be explained, at least in part, by the presence of CSCs [6]–[8], [34]. Consequently, the development of a reliable model of CSCs becomes crucial for basic and clinical cancer research.

Several techniques have been used to isolate CSCs from cancers (Table 2 and Figure S1). Initially, as the specific surface markers CD34 and CD38 had been extensively validated in the identification of normal hematopoietic stem cells, these molecules were used as markers in the original studies of leukemia stem cells [28]. Subsequently, CD24 and CD44 were selected as CSC markers in breast tumors [29]. Nevertheless, currently there is no apparent consensus regarding the “best marker(s)” to be used for the identification of CSCs in any particular cancer. There are some reports demonstrated that CD44 is a selective marker of CSCs from HNSCC [19], [20]. However, our data showed that CD24 and CD44 were abundantly present in both parental cells and spheres (up to 20–40%) (Figure S2). We selected two other representative stem cell surface markers of OSCC, CD133 and ALDH1, to detect the expression profile of both parental cells and spheres [11], [14]–[18]. The expression of CD133 and ALDH1 was usually absent or very low in parental cells compared with higher CD133 (3–4%) and ALDH1 (20–30%) expression in spheres. Although the expression of CD133 and ALDH1 was significantly higher in spheres than in parental cells, CD133 and ALDH1 were relatively adequate CSC markers in OSCC, but were not appropriate for the isolation of CSCs from the cancer proper because of tumor heterogeneity and unpredictable reproducibility (Figure S1A). The identification of specific surface marker(s) for the identification of CSCs and therapeutic targets remains a challenge. Sorting the side populations of cancer cells via intracellular Hoechst 33342 exclusion and/or selecting the chemotherapeutic-drug-resistant cells have also been used for the identification and characterization of CSCs [21]–[23], [31]. However, the method of sorting the side populations via Hoechst 33342 exclusion yielded only a small number of CSCs (0.23–22.3%), which is inadequate for further experimentation [21], [22], [31]. Recent studies showed that CSC selection via isolation of chemotherapeutic-drug-resistant cells can provide a limited number of CSCs (20–40%); however, the production of larger amounts of CSCs was expensive and time consuming (Figure S1B) [17]. Recent studies have also suggested that CSCs can be enriched in spheres when cultured in serum-free medium supplemented with adequate growth factors [11], [24]–[26]. The production of CSCs derived from OSCC cells cultured in serum-free medium supplemented with bFGF and EGF was a long, time-consuming, and cost-ineffective procedure for sphere formation [11], as shown in the upper part of Figure S1C.

**Table 2:** Comparison of the techniques in terms of isolation of CSCs related to time, cost, quantity and morphology.

Method	Time	Cost	Efficiency of isolated CSC	Morphology of isolated CSC	References
sorting by specific CSCs surface markers	nil	moderate	few(2.5∼50%)	nil	[14]–[20], [33]
sorting by side population cells	nil	moderate	few(0.23∼22.3%)	nil	[21], [22]
selecting by chemotherapeutic drug	4 weeks	moderate	moderate(20∼40%)	nil	[23]
sphere culture via serum free medium with growth factor	2∼6 weeks	high	Many(immeasurable)	sphere-like bodies	[11], [40]
sphere culture via serum free medium with growth factor	10 to 15 days	high	Many(immeasurable)	grapes-like bodies	[24], [26]
**sphere culture via non-adhesive culture system**	**5 to 7 days**	**economic**	**Abundant** **(80 to 90%)**	**sphere formation**	**current study**

Previous studies revealed that many types of cells have been described regarding the formation of 3D spheroids when cultured in suspension or in a nonadhesive environment [35], [36]. 3D spheroids are widely used as study models for cancer metastasis and invasion and for therapeutic screening; however, to the best to our knowledge, none of them mentioned the properties of CSCs [36]–[39]. In the current study, we first established a model of rapid and adequate sphere formation from human OSCC cell lines. Based on a nonadhesive culture system, this model was time efficient because spheres were generated within 5 to 7 days (Figure 1A). In addition, this modified nonadhesive culture system is cost-effective and does not require growth factors compared with the previous sphere culture system. It can not only successfully enrich sphere formation from OSCC cell lines (SAS, OECM-1, Cal27, SCC25, and Ca922), but also generates spheres from cancer cell lines from other parts of the head and neck (Fadu and TW205), from the colon (HT29 and COLO320), and from the lung (NCI-H23 and NCI-H661) (data not shown). Certain evidence shows that sphere formation can be reached within 10–15 days in serum-free medium supplemented with growth factors [30], [40]. However, these spheres are morphologically more likely to be aggregates of grape-like bodies with irregular contour, and not really spheres, as those seen in our study (Table 2 and Figure S1). In our nonadhesive culture system, the spheres appeared more tightly attached, ball-like, round, and smooth in contour. Furthermore, the expression of representative cancer stem cell genes and related proteins, including *SOX2*, *Oct4*, and *NANOG*, was upregulated in spheres compared with those detected in parental cells, at both the RNA and protein levels (Figures 3A and B). Using immunofluorescence analysis, we demonstrated that spheres exhibit explicit histological heterogeneity, as well as CSC properties (Figure 3C). Evidence of enhanced therapeutic resistance by CSCs, which is another major property of these cells, has been reported. The phenomenon of recurrence of many cancers after chemo- or radiotherapy can result from the survival and maintenance of CSCs. In our study, we demonstrated that spheres were more radio- and chemoresistant compared with parental cells (Figure 4A and B). Because of the different origin and characteristics of SAS and OECM-1 cells, there was a different treatment outcome in these two types of cells. SAS cells were more sensitive to chemotherapy, but more resistant to radiation; in contrast, OECM-1 cells were more sensitive to radiation, but more resistant to chemotherapy. CCRT was more effective in reducing survival rate for both parental cells and spheres compared with a single treatment with either radiation or chemotherapy. Nevertheless, spheres were still more resistant than parental cells when using the combined treatment. The use of this nonadhesive culture system may provide a new insight and a new model of CSCs that is applicable in therapeutic research. Xenotransplantation studies can also help identify and confirm the consecutively tumorigenic capability of nonadhesive culture systems. Inoculation of both parental cells and spheres in NOD-SCID mice generated new tumor(s) 7 days after implantation and led to an increase in tumor size over time. A comparative analysis showed that sphere-generated tumors exhibited a much larger size with an irregular, expansible contour compared with those generated by parental cells (Figure 5A). Based on primary culture of the dissolved cells of sphere-generated tumors, which were processed using the same protocols, primary and secondary spheres were generated successfully, indicating their capacity for self-renewal (Figure 5C). Interestingly, the corresponding histological and immunohistochemical results showed that tumors derived from spheres exhibited a loss of E-cadherin and upregulation of fibronectin, appeared to be more aggressive, and had a mesenchymal-like appearance compared with tumors derived from parental cells (Figure 5B).

As mentioned earlier, the enriched spheres cultured from OSCC cell lines via a nonadhesive culture system may initially become suspended and detached from the parental cells, and form small clusters. Such spheres grown in a nonadhesive condition subsequently exhibit reduced cell–cell or cell–matrix interactions, lose their anchorage, and became homeless. This triggers a phenomenon called “anoikis,” presumably resulting in apoptotic response [41]. Floating spheres in a state of anoikis in the culture medium are isolated and, although they attempt to adhere, are unable to attach to the underlying or surrounding plate which are expected to vanish in the end. How can these cancer cells survive and proliferate to overcome the threat of anoikis? What mechanism is involved in the acquisition of survival signals that offer the ability to survive and proliferate in a floating tumor population that lacks the normal solid-phase scaffolding, which constitutes a challenged microenvironment? Several studies have suggested that the adversity met by spheres in a nonadhesive, suspended condition can be stimulated by EMT and also encourage the enrollment of the potential of CSC properties [42], [43]. The literature also reveals that some signaling pathways mediate EMT and CSC properties, such as WNT, Sonic hedgehog, Snail/Slug, and NOTCH [44]–[46]. There is increasing evidence suggesting that a link exists between EMT and CSCs that involves cell morphology alteration and motility. These concepts explain why our nonadhesive culture system can be used to enrich CSCs from cancer cell lines.

In conclusion, using a modified nonadhesive culture system and a subsequent series of experiments, we not only validated the CSC properties of spheres isolated from OSCC cell lines, but also successfully established a rapid and economic method that can provide new insights and a newly applicable model for CSC research.

## Materials and Methods

### Cells

The human tongue cancer cell line SAS, obtained from the Japanese Collection, was cultured in DMEM supplemented with 10% fetal bovine serum (FBS) at 37°C in the presence of 5% CO_2_. The human gingival squamous carcinoma cell line with a p53 missens OECM-1, was cultured in RPMI1640 supplemented with 10% FBS at 37°C in the presence of 5% CO_2_. These two well established cell lines were kindly provided by the Dr. Yi-Shing Shieh from Department of Oral Diagnosis and Pathology, Tri-Service General Hospital, Taipei, Taiwan [47].

### Sphere culture

The two cell lines were cultured in culture plastic wares with nonadhesive surface. 10 cm dish are made of nonadhesive for cells by coating with agarose thin films. Cells were plated at a density of 5×10^4^ live cells/10 cm dish, and the culture medium was changed every other day until the sphere formation.

### Immunohistochemistry

Tissue sections or cell block were de-waxed in xylene and rehydrated in alcohol. Antigen retrieval was carried out by incubation in 10 mM citrate buffer (pH 6.0) at 95°C for 40 min. Endogenous peroxidase was blocked with 0.3% hydrogen peroxide for 10 min then incubated with 5% normal horse serum in phosphate-buffered saline (PBS) for 60 min at room temperature to block non-specific antibody reaction. After a wash with Tris-buffered saline plus 0.1% Tween 20(TBST), slides were incubated overnight at 4°C with primary antibodies, E-cadherin(sc-8426; 1∶800) and fibronetin (sc-18825; 1∶500) (Santa Cruz Biotechnology, Inc., CA. USA). After being rinsed in TBST, slides were incubated for 30 min at room temperature with biotinylated secondary antibody followed by streptavidin–biotinylated–enzyme complex (streptABComplexes kit; Dako, Glostrup, Denmark). Subsequently, they were stained with 0.003% 3,3-diaminobenzidine tetrahydrochloride, counterstained with Mayer's hematoxylin, dehydrated, and mounted.

### Flow cytometry

1×10^6^ single-cell suspension from trypsinized cells and spheres were responded in 1 ml PBS and stained with CD133 (clone C24B9, 1∶200) (Cell Signaling Technology, Danvers, MA) and aldehyde dehydrogenase 1 (ALDH1) (ALDEFLUOR assay kit; StemCell Technologies, Durham, NC, USA). After labeling, the cells were washed with PBS three times and subsequently stained with FITC- or PE-labeled secondary antibody for 30 min in the dark. The cells were analyzed on a flow cytometer after three washes with PBS.

### Reverse transcription-polymerase chain reaction (RT-PCR)

Total RNA was isolated with TRIzol Reagent (Invitrogen, Carlsbad, California, USA) and quantified by spectrophotometry at 260 nm. On a GeneAmp® PCR System 9700 thermocycler (Applied Biosystems, Foster City, CA, USA), 5 µg of each total RNA was reverse transcribed with SuperScript III (Invitrogen) at 55°C for 1 hour into total complementary DNA, which was used as the template for the subsequent PCR reactions and analysis. The PCR reactions involved an initial denaturation at 94°C for 5 minutes, followed by 25 or 30 cycles at 94°C for 30 seconds, exposure to an appropriate annealing temperature (58–62°C) for 30 seconds, and then a final incubation at 72°C for 45 seconds. The PCR primers for analysis of mRNA were: Glyceraldehyde-3-phosphate dehydrogenase (GAPDH), sense (5′-AGCCGCATCTTCTTTTGCGTC-3′) and antisense (5′-TCATATTTGGCAGGTTTTTCT-3′);

Oct-4, sense (5′-CGCACCACTGGCATTGTCAT-3′)

and antisense (5′-TTCTCCTTGATGTCACGCAC-3′);

Nanog, sense (5′-AATACCTCAGCCTCCAGCAGATG-3′)

and antisense (5′-CTGCGTCACACCATTGCTATTCT-3′);

SOX2, sense (5′-GGCAGCTACGCATGATGCAGGAGC-3′)

and antisense (5′-CTGGTCATGGAGTTGTACTGCACG-3′). Amplified RT-PCR products were then analyzed on 1% agarose gels and visualized using ethidium bromide staining and a camera system (Transilluminator/SPOT; Diagnostic Instruments, Sterling Heights, MI, USA). The gel images of the RT–PCR products were directly scanned (ONEDscan 1-D Gel Analysis Software; Scanalytic Inc. Fairfax, VA, USA), and the relative densities were obtained by determining the ratio of the signal intensity to the GAPDH band. Gene expression between the test (cyclosporine A treated) and the control groups was compared.

### Western blotting

Whole cell lysates were separated by electrophoresis on 12% SDS–PAGE and transferred to polyvinylidene fluoride membrane. The membranes were blocked with 5% nonfat milk at room temperature for 1 h. The primary antibodies were used: GAPDH (ab9482; 1∶5000 dilution) (Abcam, Cambridge, MA, USA), Oct-3/4 (sc-8630; 1∶1000), NANOG (sc-81961; 1∶1000) and SOX2 (sc-17320; 1∶500) (Santa Cruz Biotechnology) in TBST buffer containing 3% nonfat milk at 4°C overnight and subsequently with anti-mouse and rabbit anti-goat secondary antibody conjugated with peroxidase (1∶1000) (Santa Cruz Biotechnology) at 25°C for 1 h. The immunoblots were developed using an enhanced chemiluminescence system, and the luminescence was visualized on X-ray film.

### Immunofluorescence

The living cells and spheres were fixed in 4% paraformaldehyde, permeabilized in 0.1% Triton X-100, and blocked in 5% normal goat serum- PBS. Cells were incubated with primary antibodies, Oct-3/4 (sc-8630, 1∶200), NANOG (sc-81961,1∶200), SOX2 (sc-17320; 1∶500) (Santa Cruz Biotechnology), CD133 (clone C24B9,1∶200) (Cell Signaling Technology) and ALDH1 (clone 44, 1∶200) (BD Biosciences, San Jose, CA, USA) washed thrice in PBS, and then incubated with goat anti-mouse or secondary antibodies conjugated with FITC (green) or PE (red). The DAPI was used as nuclear stain (blue). Images were obtained using fluorescent microscopy and a digital camera.

### Chemosensitivity and radiosensitivity assay

Cells were seeding in 10 cm dish at a density of 1×10^6^ cells/dish. For the chemosensitivity assay, cells were treated with 10–200 µM Cisplatin (Sigma, St Louis, MO, USA) for 48 h. For the radioresistance assay, cells were irradiated using a CyberKnife radiosurgery system (Accuray, USA) to deliver different doses (2–10 Gy). Relative survival fraction of cells was determined by MTS assay using the CellTiter 96 Aqueous One Solution Cell Proliferation Assay kit (Promega, Madison, WI, USA) after 36 h of radiation treatment.

### *In vivo* tumorigenicity study

The in vivo tumorigenicity study was performed following local ethics committee guidelines that had full accreditation awarded by the Association for Assessment and Accreditation of Laboratory Animal Care in the National Defense Medical Center. Mice were kept at 18–26°C, 30–70% humidity, and independently air-conditioned under a 12 h dark/12 h light cycle for 7 days before xenograft injection. The parental OSCC cells and spheres were injected into the BALB/c nude mice (6 weeks). The cell suspension (100 µl) was injected subcutaneously in each mouse with different cell numbers from 1×10^6^, 1×10^5^, 1×10^4^ cells. Tumors were formed in 7 days after injection. Tumor sizes were monitored and measured weekly according to the formula: (length×width^2^)/2. At 30 days after orthotopic inoculation, mice were euthanized under anesthesia. All of the animals were conformed and approved by the Institutional Animal Care and Use Committee in National Defense Medical Center (IACUC-11-064).

### Statistical analysis

The independent Student's t test or ANOVA was used to compare the continuous variables between groups, whereas the Χ^2^ test was applied for the comparison of dichotomous variable. The level of statistical significance was set at 0.05 for all tests. All statistical analyses were performed using SPSS version 12.0 (SPSS, Inc., Chicago, IL, USA).

## Supporting Information

Figure S1 **Diagrammatic illustration of the comparison of the techniques used for the isolation of CSCs.** (A) Isolation of CSCs using surface CSC markers. (B) Alternative option of CSC isolation via sorting of side population cells and/or selection of chemotherapeutic-drug-resistant cells. (C) Comparison of sphere formation in terms of time and morphology between sphere culture using serum-free medium with growth factors (upper panel) and sphere culture using a nonadhesive system (lower panel).
(DOCX)

Figure S2 **Comparison of the expressions of CD24 and CD44 between parental cells and spheres.** The parental cells and spheres were either stained with a negative-control IgG antibody (open space), (A) anti-CD24 or (B) anti-CD44 experimental antibodies (solid space). (C) CD24 and CD44 were abundantly present in both parental cells and spheres; there is no significant difference between these two groups.
(DOCX)

## References

[1] Jemal A, Bray F, Center MM, Ferlay J, Ward E (2011). Global cancer statistics.. CA Cancer J Clin.

[2] Al-Swiahb JN, Chen CH, Chuang HC, Fang FM, Tasi HT (2010). Cinical, pathological and molecular determinants in squamous cell carcinoma of the oral cavity.. Future Oncol.

[3] Olasz L, Szabo I, Horvath A (1988). A combined treatment for advanced oral cavity cancers.. Cancer.

[4] Lippman SM, Sudbo J, Hong WK (2005). Oral cancer prevention and the evolution of molecular-targeted drug development.. J Clin Oncol.

[5] Le Tourneau C (2010). Molecularly targeted therapy in head and neck cancer.. Bull Cancer.

[6] Clarke MF, Dick JE, Dirks PB, Eaves CJ, Jamieson CH (2006). Cancer stem cells – Perspectives on current status and future directions: AACR Workshop on Cancer Stem Cells.. Cancer Res.

[7] Jordan CT, Guzman ML, Noble M (2006). Cancer stem cells.. N Engl J Med.

[8] Dalerba P, Cho RW, Clarke MF (2007). Cancer stem cells: models and concepts.. Annu Rev Med.

[9] Costea DE, Tsinkalovsky O, Vintermyr OK, Johannessen AC, Mackenzie IC (2006). Cancer stem cells new and potentially important targets for the therapy of oral squamous cell carcinoma.. Oral Dis.

[10] Locke M, Heywood M, Fawell S, Mackenzie IC (2005). Retention of intrinsic stem cell hierarchies in carcinoma-derived cell lines.. Cancer Res.

[11] Chiou SH, Yu CC, Huang CY, Lin SC, Liu CJ (2008). Positive correlations of Oct-4 and Nanog in oral cancer stem-like cells and high-grade oral squamous cell carcinoma.. Clin Cancer Res.

[12] Dean M, Fojo T, Bates S (2005). Tumor stem cells and drug resistance.. Nat Rev Cancer.

[13] Jordan CT, Guzman ML (2004). Mechanisms controlling pathogenesis and survival of leukemic stem cells.. Oncogene.

[14] Wei XD, Zhou L, Cheng L, Tian J, Jiang JJ (2009). In vivo investigation of CD133 as a putative marker of cancer stem cells in Hep-2 cell line.. Head Neck.

[15] Zhang Q, Shi S, Yen Y, Brown J, Ta JQ (2010). A subpopulation of CD133(+) cancer stem-like cells characterized in human oral squamous cell carcinoma confer resistance to chemotherapy.. Cancer Lett.

[16] Zhou L, Wei X, Cheng L, Tian J, Jiang JJ (2007). CD133, one of the markers of cancer stem cells in Hep-2 cell line.. Laryngoscope.

[17] Clay MR, Tabor M, Owen JH, Carey TE, Bradford CR (2010). Single-marker identification of head and neck squamous cell carcinoma cancer stem cells with aldehyde dehydrogenase.. Head Neck.

[18] Chen YC, Chen YW, Hsu HS, Tseng LM, Huang PI (2009). Aldehyde dehydrogenase 1 is a putative marker for cancer stem cells in head and neck squamous cancer.. Biochem Biophys Res Commun.

[19] Shen S, Yang W, Wang Z, Lei X, Xu L (2011). Tumor-initiating cells are enriched in CD44(hi) population in murine salivary gland tumor.. PLoS One.

[20] Prince ME, Sivanandan R, Kaczorowski A, Wolf GT, Kaplan MJ (2007). Identification of a subpopulation of cells with cancer stem cell properties in head and neck squamous cell carcinoma.. Proc Natl Acad Sci U S A.

[21] Yanamoto S, Kawasaki G, Yamada S, Yoshitomi I, Kawano T (2011). Isolation and characterization of cancer stem-like side population cells in human oral cancer cells.. Oral Oncol.

[22] Song J, Chang I, Chen Z, Kang M, Wang CY (2010). Characterization of side populations in HNSCC: highly invasive, chemoresistant and abnormal Wnt signaling.. PLoS One.

[23] Tsai LL, Yu CC, Chang YC, Yu CH, Chou MY (2011). Markedly increased Oct4 and Nanog expression correlates with cisplatin resistance in oral squamous cell carcinoma.. J Oral Pathol Med.

[24] Lee J, Kotliarova S, Kotliarov Y, Li A, Su Q (2006). Tumor stemcells derived from glioblastomas cultured in bFGF and EGF more closely mirror the phenotype and genotype of primary tumors than do serum-cultured cell lines.. Cancer Cell.

[25] Hueng DY, Sytwu HK, Huang SM, Chang C, Ma HI (2011). Isolation and characterization of tumor stem-like cells from human meningiomas.. J Neurooncol.

[26] Zhong Y, Guan K, Guo S, Zhou C, Wang D (2010). Spheres derived from the human SK-RC-42 renal cell carcinoma cell line are enriched in cancer stem cells.. Cancer Lett.

[27] Chen C, Wei Y, Hummel M, Hoffmann TK, Gross M (2011). Evidence for epithelial-mesenchymal transition in cancer stem cells of head and neck squamous cell carcinoma.. PLoS One.

[28] Bonnet D, Dick JE (1997). Human acute myeloid leukemia is organized as a hierarchy that originates from a primitive hematopoietic cell.. Nat Med.

[29] Al-Hajj M, Wicha MS, Benito-Hernandez A, Morrison SJ, Clarke MF (2003). Prospective identification of tumorigenic breast cancer cells.. Proc Natl Acad Sci U S A.

[30] Ponti D, Costa A, Zaffaroni N, Pratesi G, Petrangolini G (2005). Isolation and in vitro propagation of tumorigenic breast cancer cells with stem/progenitor cell properties.. Can Res.

[31] Sung JM, Cho HJ, Yi H, Lee CH, Kim HS (2008). Characterization of a stem cell population in lung cancer A549 cells.. Biochem Biophys Res Commun.

[32] Lukacs RU, Memarzadeh S, Wu H, Witte ON (2010). Bmi-1 is a crucial regulator of prostate stem cell self-renewal and malignant transformation.. Cell Stem Cell.

[33] Ricci-Vitiani L, Lombardi DG, Pilozzi E, Biffoni M, Todaro M (2007). Identification and expansion of human colon-cancer-initiating cells.. Nature.

[34] Alison MR, Lim SM, Nicholson LJ (2010). Cancer stem cells: problems for therapy?. Pathol.

[35] Lin RZ, Chang HY (2008). Recent advances in three-dimensional multicellular spheroid culture for biomedical research.. Biotechnol J.

[36] Mueller-Klieser W (1997). Three-dimensional cell cultures: from molecular mechanisms to clinical applications.. Am J Physiol.

[37] Kunz-Schughart LA, Freyer JP, Hofstaedter F, Ebner R (2004). The use of 3-D cultures for high-throughput screening: The multicellular spheroid model.. J Biomol Screen.

[38] Dubessy C, Merlin JM, Marchal C, Guillemin F (2000). Spheroids in radiobiology and photodynamic therapy.. Crit Rev Oncol Hematol.

[39] Timmins NE, Dietmair S, Nielsen LK (2004). Hanging-drop multicellular spheroids as a model of tumor angiogenesis.. Angiogenesis.

[40] Yu SC, Ping YF, Yi L, Zhou ZH, Chen JH (2008). Isolation and characterization of cancer stem cells from a human glioblastoma cell line U87.. Cancer Lett.

[41] Liotta LA, Kohn E (2004). Anoikis: cancer and the homeless cell.. Nature.

[42] Avizienyte E, Fincham VJ, Brunton VG, Frame MC (2002). Src-induced de-regulation of E-cadherin in colon cancer cells requires integrin signaling.. Nat Cell Biol.

[43] Fujita Y, Krause G, Scheffner M, Zechner D, Leddy HE (2002). Hakai, a c-Cbl-like protein, ubiquitinates and induces endocytosis of the E-cadherin complex.. Nat Cell Biol.

[44] Huber MA, Kraut N, Beug H (2005). Molecular requirements for epithelial–mesenchymal transition during tumor progression.. Curr Opin Cell Biol.

[45] Bailey JM, Singh PK, Hollingsworth MA (2007). Cancer metastasis facilitated by developmental pathways: Sonic hedgehog, Notch, and bone morphogenic proteins.. J Cell Biochem.

[46] Ouyang G, Wang Z, Fang X, Liu J, Yang CJ (2010). Molecular signaling of the epithelial to mesenchymal transition in generating and maintaining cancer stem cells.. Cell Mol Life Sci.

[47] Lee CH, Hung HW, Hung PH, Shieh YS (2010). Epidermal growth factor receptor regulates beta-catenin location, stability, and transcriptional activity in oral cancer.. Mol Cancer.

