# Supplementary figures and images for: Nonadhesive Culture System as a Model of Rapid Sphere Formation with Cancer Stem Cell Properties
Source: PLoS One. 2012 Feb 16;7(2):e31864. doi: 10.1371/journal.pone.0031864 (PMC3281010; doi:10.1371/journal.pone.0031864)

**Supporting information figure S1**

**
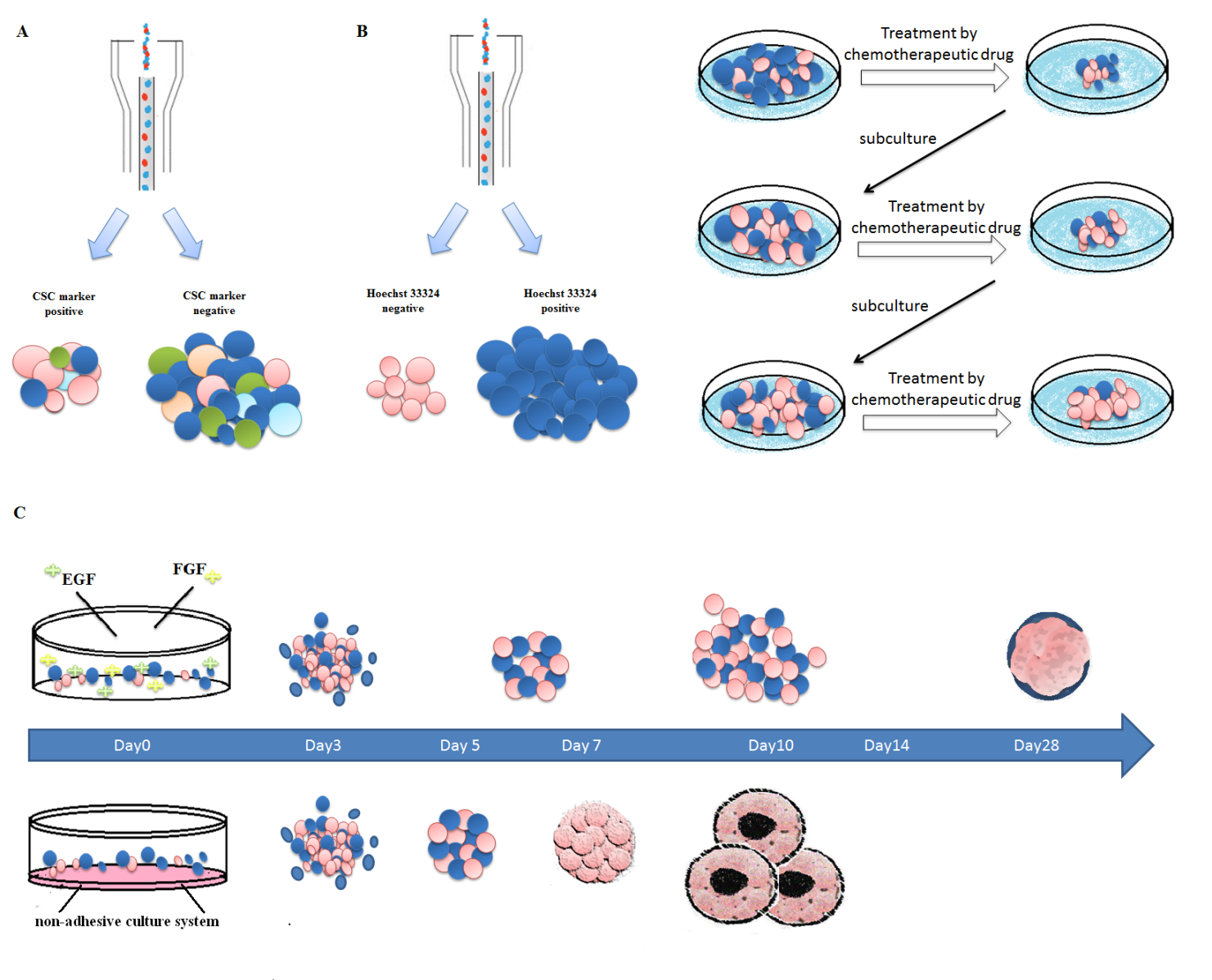
**

Supplement: Figure S1 — Diagrammatic illustration of the comparison of the techniques used for the isolation of CSCs. (A) Isolation of CSCs using surface CSC markers. (B) Alternative option of CSC isolation via sorting of side population cells and/or selection of chemotherapeutic-drug-resistant cells. (C) Comparison of sphere formation in terms of time and morphology between sphere culture using serum-free medium with growth factors (upper panel) and sphere culture using a nonadhesive system (lower panel). (DOCX) [file pone.0031864.s001.docx]

**Supporting information figure S2**


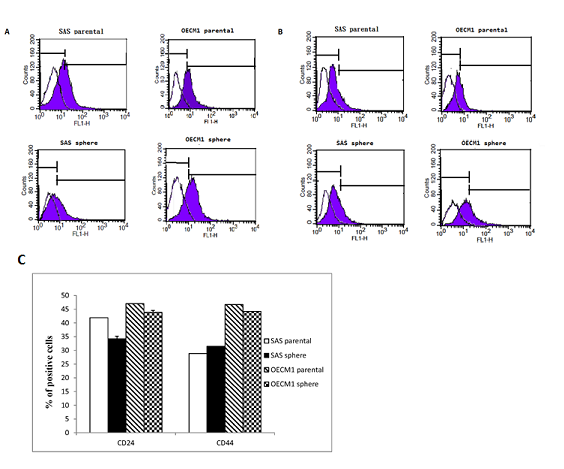

Supplement: Figure S2 — Comparison of the expressions of CD24 and CD44 between parental cells and spheres. The parental cells and spheres were either stained with a negative-control IgG antibody (open space), (A) anti-CD24 or (B) anti-CD44 experimental antibodies (solid space). (C) CD24 and CD44 were abundantly present in both parental cells and spheres; there is no significant difference between these two groups. (DOCX) [file pone.0031864.s002.docx]
